# Measurement invariance of the Depression Anxiety Stress Scales-21 across medical student genders

**DOI:** 10.5116/ijme.58ba.7d8b

**Published:** 2017-03-30

**Authors:** Peyman Jafari, Farnoosh Nozari, Forooghosadat Ahrari, Zahra Bagheri

**Affiliations:** 1 Department of Biostatistics, Shiraz University of Medical Sciences, Shiraz, Iran; 2 Student Research Committee, Shiraz University of Medical Sciences, Shiraz, Iran

**Keywords:** Measurement invariance, medical students, DASS-21, Iran

## Abstract

**Objectives:**

This study aimed to assess whether male and female Iranian medical students perceived the meaning of the items in the Depression Anxiety Stress Scales-21 consistently.

**Methods:**

A convenience sample of 783 preclinical medical students from the first to sixth semester was invited to this cross-sectional study. Of the 477 respondents, 238 were male and 239 were female. All participants completed the Persian version of the Depression Anxiety Stress Scales-21. The graded response model was used to assess measurement invariance of the instrument across the gender groups. Categorical confirmatory factor analysis was used to evaluate the construct validity of the measure. Moreover, internal consistency was assessed via Cronbach's Alpha.

**Results:**

Statistically significant differential item functioning was flagged for just item 6 in the depression subscales (c^2^=6.5, df=1, p=0.011). However, removing or retaining the item 6 in the stress subscale did not change our findings significantly, when we compared stress scores across two genders. The results of categorical confirmatory factor analysis supported the fit of the three-factor model of Depression Anxiety Stress Scales-21. Moreover, Cronbach’s alpha was greater than 0.7 in depression, anxiety and stress subscales.

**Conclusions:**

This study revealed that Depression Anxiety Stress Scales-21 is an invariant measure across male and female medical students. Hence, this reliable and valid instrument can be used for meaningful comparison of distress scores between medical student genders. Gender comparisons of medical students’ psychological profiles provide a better insight into gender influences on the outcome of medical education and medical practice.

## Introduction

Medical education is a long process where students face multiple stressors such as academic pressure, workload, sleep deprivation, emotional pressure to maintain good grades, lack of leisure time, and sometimes financial concerns. Every year hundreds of thousands of Iranian high school graduates compete in the extremely difficult and challenging exam, and only less than 3,000 among them are admitted to the public-funded medical schools around the country.^1^ The medical education programme in Iran takes a minimum of seven years; it includes basic science period or preclinical stage, physiopathology period (theoretical aspects of different common diseases), and internship period during which the students practice at university hospitals and work under the supervision of residents and fully licensed staff physicians. According to previous research, medical students in Iran^2^ and in other countries^3^^,^^4^ are prone to experiencing high levels of depression, anxiety, and stress during their training. These studies have shown that these students have higher psychological distress than the general population.^5^^-^^9^ A systematic review, which was restricted to medical schools in Europe and the English-speaking world outside North America, reported that rate of anxiety, depression, and psychological distress in medical students varies from 7.7% to 65.5%, 6.0% to 66.5%, and 12.2% to 96.7%, respectively.^4^

In order to reduce distress in medical students and develop a training programme to produce the best possible physicians, medical educators must consider gender differences as one of the most important demographic factors existing in the medical student population.^10^ Gender differences have been evaluated across medical students to explore how they experience and cope with distress as well as what they think about the role of gender in distress.^2^^,^^11^^-^^20^ According to literature reviews,^3^^,^^4^ female medical students reported higher levels of depression, anxiety, and stress than their male peers.^12^^-^^16^ In contrast, a number of other studies reported either no difference between the genders^2^^,^^11^^,^^17^ or higher levels of distress in male students.^18^^,^^19^ These discrepancies relating to gender in previous research may have other origins and should be interpreted with caution.

It has been recognized that psychological measurements are sensitive to individual characteristics such as age and gender groups.^21^ Accordingly, researchers should be confident that the items comprising the distress questionnaires are equivalently interpreted by male and female medical students when they intend to compare distress scores between the two groups. This issue defined as measurement invariance is a prerequisite assumption for psychological comparisons across different groups (e.g. gender). Measurement invariance, also known as differential item functioning (DIF) analysis, evaluates whether the probability of responding to a specific item within a measure is the same across the compared groups after controlling for the certain construct.^22^ If this assumption does not hold, the comparison of distress scores across male and female medical students are not valid and differences between groups cannot be meaningfully interpreted. This is because differences in distress scores across gender groups must represent true differences in the construct of interest and not reflect the measurement bias. In previous studies, a variety of instruments have been used to assess depression, anxiety, and stress between male and female university students.^4^^,^^10^^-^^14^^,^^19^^,^^20^^,^^23^^-^^25^ One of the most widely accepted instruments for assessing the severity of distress in clinical and non-clinical samples is Depression Anxiety Stress Scale-21 (DASS-21).^26^^-^^37^ Although measurement invariance of the scale is evaluated across racial groups, and between male and female with chronic low back pain, such an explanation has never been provided across gender in medical students.^29^^,^^38^ As far as we know, there are just three studies that have recently examined DIF across male and female students through a multiple-group confirmatory factor analysis (MGCFA) in the Beck Depression Inventory (BDI), General Health Questionnaire (GHQ-12) and College Student Stress Scale (CSSS) instruments.^39^^-^^41^ However, since non-medical students participated in these three studies, the generalizability of the findings with regard to medical students has remained ambiguous. To fill this gap, the present DIF study is designed to assess whether male and female Iranian medical students perceive the meanings of the items in the DASS-21 consistently. Accordingly, this study addresses whether distress scores extracted from the DASS-21 are comparable across gender in Iranian medical students.

## Methods

### Study design and participants

This cross-sectional study has been conducted over the first- to sixth-semester medical students who began their medical training between 2012 and 2015 academic years at Shiraz University of Medical Sciences. Shiraz, Iran. A convenience sample of 783 preclinical medical students (399 male, 384 female) were invited to participate into the study from October to December 2015; among them 477 students (238 male, 239 female) accepted to enter the study. The study was approved by the ethics committee of the university.

### Procedure

Two trained medical students distributed the Persian version of the DASS-21 instrument along with a consent form to preclinical medical students in each semester before starting some specific mandatory classes. The students who intended to participate into the study signed the consent form, completed the Persian versions of the DASS-21 and submitted them individually to one of the distributers to ensure confidentiality.

### Data collection

The English version of the DASS-21 questionnaire was translated into Persian by using standard guidelines, including independent forward and back translation. The finalized Persian version of the measure was very similar to those used in the last two previous studies.^42^^,^^43^ They reported that the Cronach’s alpha coefficients for the three DASS-21 subscales varied from 0.85 to 0.87 and from 0.81 to 0.98, in clinical and non-clinical Iranian samples, respectively.^42^^,^^43^ The DASS questionnaire is in public domain and so no permission was needed to use it. This 21-item questionnaire contains three subscales including depression (seven items), anxiety (seven items), and stress (seven items). The students responded to the items on a 4-point Likert scale (0 = never a problem, 1=sometimes a problem, 2=often a problem, and 3 =almost always a problem). According to the DASS-21 scoring algorithm, higher scores indicated higher depression, anxiety and stress. Total score is calculated by summing the scores for each subscale. Moreover, DASS scoring manual have provided cut-off scores for defining normal (0-4 for depression, 0-3 for anxiety and 0-7 for stress), mild (5-6 for depression, 4-5 for anxiety and 8-9 for stress), moderate (7-10 for depression, 6-7 for anxiety and 10-12 for stress), severe (11-13 for depression, 8-9 for anxiety and 13-14 for stress) and extremely severe (>14 for depression, >10 for anxiety, >17 for stress) scores.

### Statistical analysis

The reliability of the DASS-21 was examined by Cronbach’s alpha coefficient. A coefficient equal to or greater than 0.7 was considered to be a satisfactory level of reliability. Convergent validity of the DASS-21 was assessed using Spearman correlation. This measure provides evidence to decide which items should be excluded from their own domain. The value of a correlation coefficient of greater than 0.40 between an item and its own subscale was regarded as an adequate evidence of convergent validity.^44^ Mean item-correlation which is the average correlations between all pairs of items in each subscales of the DASS-21 was also computed. It provides an index for the assessment of item redundancy showing that to what extent items on a certain subscale measure the same content. Ideally, mean item-correlation for a set of items should be between 0.20 and 0.40. Values less than 0.2 indicate that the items may not be representative of the same construct. If values, on the other hand, are higher than 0.4, the items may capture only a small bandwidth of the construct.^45^

In order to evaluate the construct validity of the questionnaire, categorical confirmatory factor analysis (CCFA) was used. Generally, CCFA investigates the relationship between a set of observed variables (the items of the DASS-21) and a set of continuous latent constructs (depression, anxiety, and stress subscales). In the present study, we investigated whether or not the hypothesized three-factor model fit the data well for the whole sample and also for each gender group. Several criteria were used to assess the goodness of fit of the model, including chi-square statistics, root mean square error of approximation (RMSEA), Tuker-Lewise index (TLI) and comparative fit index (CFI). Since chi-square statistics are known to be sensitive to large samples, this test may not be a realistic fit index, and therefore, the other above-mentioned fit indices were considered for assessing goodness of fit of the model.^46^ Values of CFI and TLI ≥ 0.90, and RMSEA ≤ 0.08 can support acceptable model fit.^47^ The mean- and variance-adjusted weighted least square (WLSMV) estimation procedure using the Mplus 6.1 software was used to perform the CCFA.

In the present study, the graded response model (GRM) was used to assess the measurement invariance of the DASS-21 across male and female Iranian medical students. Two different types of DIF, uniform and non-uniform, can be distinguished by GRM.^48^ Uniform DIF occurs when the difference in an item’s response probabilities is constant along the complete construct continuum scale between two groups (i.e., threshold parameters are statistically different). In non-uniform DIF, the direction of the DIF differs along the construct scale, meaning that there is interaction between the construct level and group membership (i.e., discrimination parameters are significantly different). This study used IRTPRO2.1 software to detect uniform and non-uniform DIF, and to estimate discrimination and threshold parameters across two samples.

## Results

Table 1 shows Cronbach’s alpha coefficients along with the results of convergent validity and mean item-correlation in each subscale of the DASS-21. All the subscales of the DASS-21 had adequate internal consistency, which was greater than 0.7. Moreover, scaling success rates for convergent validity were 100% in all domains with the exception of the stress subscale. In the stress subscale, the total stress score for the seven items was calculated and used as a new variable in the analysis. Then the correlations (r) between individual items and the total stress score were computed. The seven items comprising the stress subscale had correlations of 0.38, 0.68, 0.67, 0.69, 0.66, 0.63 and 0.67 respectively with the total score of the subscale. Accordingly, six out of the seven (86%) items had a highly correlation (r = 0.4 or greater) with their own domain. In addition, as shown in Table 1, mean item-correlations within each subscale were in the acceptable ranges which support the hypothesis that the items in each domain measure the same construct.

**Table 1:** Cronbach’s alpha, convergent validity and mean item-correlation for the DASS-21 subscales

DASS subscales	Items	Cronbach’s Alpha	Convergent validity		Mean item- correlation
			Range of correlation	Scaling success (%)	
Depression	7	0.86	0.56-0.77	7/7 (100%)	0.28
Anxiety	7	0.76	0.50-0.67	7/7 (100%)	0.41
Stress	7	0.79	0.38-0.69	6/7 (86%)	0.31

Table 2 presents the values of goodness of fit indices for the three-factor CCFA model of the DASS-21 in the whole sample and each gender group. As indicated, all values of CFI and TLI were greater than 0.90 and those of RMSEA were less than 0.08 which supported the fit of the three-factor CCFA model in the whole sample and also in the male and female medical students, separately. This result confirmed the construct validity of the instrument.

**Table 2:** Goodness of fit indices for the three-factor CCFA model of the DASS-21 in the total sample and each gender group

	c^2^(df), p	CFI	TLI	RMSEA
Total sample	615.99 (186), <0.001	0.94	0.93	0.070
Female	371.63 (186), <0.001	0.96	0.95	0.065
Male	411.28 (186), <0.001	0.92	0.91	0.071

Table 3 shows the results of the estimated threshold (b_i_) and discrimination (a_i_) parameters of the GRM for assessing DIF across male and female Iranian medical students in all subscales. Items constrained to be equal across the two groups serve as anchor while items suspected of DIF (i.e., study items) are allowed to freely vary. Anchors items are not identified as potentially exhibiting uniform or non-uniform DIF and they have been previously detected in the rigorous analysis. The last two columns of Table 3 list the chi-square values (χ^2^), degrees of freedom (df) and p-values for the uniform and non-uniform DIF tests for all items in the three subscales.

**Table 3:** Item parameters and standard errors (SE) for anchor and study items used in the analysis of differential item functioning on the DASS-21 for male and female medical students using GRM

Items		Group	a(SE)	b_1_(SE)	b_2_(SE)	b_3_(SE)	Test for DIF	
							Non-uniform c^2^(df), p	Uniform c^2^(df), p
Depression								
	1. I couldn't seem to experience any positive feeling at all	Male Female	1.69(0.19) 1.69(0.19)	-0.04(0.10) -0.04(0.10)	1.53(0.15) 1.53(0.15)	2.59(0.25) 2.59(0.25)	Anchor	
	2. I found it difficult to work up the initiative to do things	Male Female	0.71(0.15) 1.09(0.19)	-1.25(0.32) -0.52(0.19)	2.08(0.46) 2.10(0.32)	4.44(0.96) 4.23(0.70)	2.5(1), 0.118	7.3(3), 0.063
	3. I felt that I had nothing to look forward to	Male Female	3.73(0.50) 3.73(0.50)	0.44(0.08) 0.44(0.08)	1.57(0.13) 1.57(0.13)	2.30(0.20) 2.30(0.20)	Anchor	
	4. I felt down-hearted and blue	Male Female	2.63(0.29) 2.63(0.29)	-0.22(0.09) -0.22(0.09)	1.33(0.12) 1.33(0.12)	2.51(0.22) 2.51(0.22)	Anchor	
	5. I was unable to become enthusiastic about anything	Male Female	1.94(0.22) 1.94(0.22)	0.38(0.09) 0.38(0.09)	1.95(0.18) 1.95(0.18)	2.96(0.29) 2.96(0.29)	Anchor	
	6. I felt I wasn't worth much as a person	Male Female	2.24(0.26) 2.24(0.26)	0.63(0.10) 0.63(0.10)	1.86(0.17) 1.86(0.17)	3.04(0.30) 3.04(0.30)	Anchor	
	7. I felt that life was meaningless	Male Female	2.42(0.28) 2.42(0.28)	0.43(0.09) 0.43(0.09)	1.59(0.15) 1.59(0.15)	2.47(0.22) 2.47(0.22)	Anchor	
Anxiety								
	1. I was aware of dryness of my mouth	Male Female	0.67(0.18) 0.73(0.15)	0.66(0.25) 0.37(0.21)	3.61(0.92) 3.79(0.72)	7.45(2.16) 6.52(1.40)	0.1(1), 0.785	3.0(3), 0.398
	2. I experienced breathing difficulty	Male Female	1.26(0.21) 1.54.(0.33)	-1.09(0.20) -1.09(0.27)	1.44(0.23) 0.70(0.25)	2.96(0.45) 2.47(0.57)	0(1), 0.85	0.6(3), 0.90
	3. I experienced trembling (e.g., in the hands)	Male Female	0.97(0.20) 1.15(0.19)	0.35(0.15) 0.53(0.16)	2.18(0.39) 2.58(0.36)	3.55(0.67) 4.41(0.68)	0.4(1), 0.532	7.0(3), 0.073
	4. I was worried about situations in which I might panic and make a fool of myself	Male Female	1.26(0.22) 1.21(0.19)	-0.23(0.13) -0.06(0.15)	1.65(0.25) 2.00(0.27)	3.02(0.48) 3.71(0.52)	0.0(1), 0.864	2.6(3), 0.453
	5. I felt I was close to panic	Male Female	2.09(0.37) 2.48(0.45)	0.67 (0.11) 0.50(0.11)	2.00(0.24) 1.84(0.19)	3.58(0.66) 2.91(0.34)	0.4(1), 0.503	1.4(3), 0.699
	6. I was aware of the action of my heart in the absence of physical exertion	Male Female	0.84(0.17) 2.00(0.42)	-0.55(0.21) -0.83(0.23)	2.18(0.42) 1.02(0.28)	3.88(0.76) 2.20(0.50)	0.2(1), 0.66	3.6(3), 0.31
	7. I felt that life was meaningless	Male Female	2.11(0.39) 1.91(0.33)	0.84(0.12) 0.75(0.12)	2.06(0.25) 2.15(0.23)	2.83(0.40) 3.08(0.37)	0.2(1) 0.696	0.7(3) 0.882
Stress								
	1. I found it hard to wind down	Male Female	0.58(0.12) 0.58(0.12)	0.66(0.23) 0.66(0.23)	4.96(1.03) 4.96(1.03)	8.04(1.78) 8.04(1.78)	Anchor	
	2. I tended to over-react to situations	Male Female	1.40(0.28) 1.33(0.23)	1.20(0.19) 1.13(0.18)	2.81(0.46) 2.88(0.38)	4.57(1.06) 4.28(0.66)	0.5(1), 0.47	6.3(3), 0.099
	3. I felt that I was using a lot of nervous energy	Male Female	1.37(0.21) 1.37(0.21)	-0.89(0.18) -0.89(0.18)	0.87(0.17) 0.87(0.17)	2.44(0.36) 2.44(0.36)	Anchor	
	4. I found myself getting agitated	Male Female	2.30(0.42) 2.73(0.56)	-0.26(0.11) -0.65(0.20)	1.44(0.16) 0.99(0.27)	2.45(0.29) 2.29(0.49)	0.4(1), 0.536	5.2(3), 0.15
	5. I found it difficult to relax	Male Female	2.22(0.40) 2.35(0.49)	-0.10(0.11) -0.61(0.20)	1.52(0.17) 1.27(0.32)	2.69(0.33) 2.11(0.47)	0.0(1), 0.834	7.6(3), 0.054
	6. I was intolerant of anything that kept me from getting on with what I was doing	Male Female	0.84(0.17) 2.00(0.42)	-0.55(0.21) -0.83(0.23)	2.18(0.42) 1.02(0.28)	3.88(0.76) 2.20(0.50)	**6.5(1), 0.011**	**10.9(3), 0.012**
	7. I felt that I was rather touchy	Male Female	1.28(0.21) 1.98(0.42)	-0.85(0.18) -1.13(0.26)	1.29(0.20) 0.45(0.21)	3.32(0.50) 1.95(0.45)	2.2(1), 0.139	6.2(3), 0.102

According to GRM, no DASS-21 items exhibited DIF across male and female medical students, except for item 6 in the stress subscale. This item displayed both uniform and non-uniform DIF, and, hence, considered as asymmetric non-uniform DIF. For item 6 in the stress subscale the threshold parameters are shifted to the right for the male students relative to the female ones. These shifts imply that female medical students with high level of stress are more likely than male counterparts with high level of stress to endorse the higher category (e.g., often or almost always a problem). Moreover, item 6 in the stress subscale is more discriminating for females than males (the a_i_ parameters are statistically different). It means that item 6 differentiates well between genders with different levels of stress.

In order to know to what extent Item 6 in the stress subscale can distort group differences (male versus female), we applied a removing and retaining strategy. As shown in Table 4, depression, anxiety, and stress scores were not statistically significant across gender medical student. Further analysis revealed that ignoring or accounting for Item 6: “I was intolerant of anything that kept me from getting on with what I was doing” with asymmetric non-uniform DIF in the stress subscale had no considerable effects on group differences.

**Table 4:** Comparison of depression, anxiety and stress rated by male and female medical students in the DASS-21

DASS subscales	Male (n=238)	Female (n=239)	t(df), p
	Mean (SD)	Mean (SD)	
Depression	4.13(3.75)	4.25(4.12)	0.34(475), 0.72
Anxiety	3.33(2.89)	3.40(3.32)	0.24(475), 0.80
Stress	5.67(3.29)	5.74(3.92)	0.22(475), 0.82
subscale corrected for DIF			
Stress^*^	4.68(2.86)	4.76(3.37)	0.26(475), 0.78

*Stress score corrected for item 6 with asymmetric non-uniform DIF

As shown in Table 5, the overall rate of depression, anxiety, and stress (including students with mild, moderate, severe, and extremely severe) found in this study was 36%, 38.6%, 25.2% and 35%, 39.7%, and 24.7% for male and female, respectively. These results showed that the rate of depression, anxiety, and stress was similar across male and female medical students.

**Table 5:** Subscale severity ratings suggested for Iranian preclinical medical students by gender

Severity ratings	Depression N (%)		Anxiety N (%)		Stress N (%)	
	Male	Female	Male	Female	Male	Female
Normal	152 (64)	155 (65)	146 (61.4)	144 (60.3)	178 (74.8)	180 (75.3)
Mild	34 (14.3)	34 (14.1)	49 (20.6)	42 (17.6)	29 (12.2)	26 (10.9)
Moderate	33 (13.9)	31 (13)	19 (8)	31 (13)	23 (9.7)	18 (7.5)
Severe	14 (5.8)	9 (3.7)	12 (5)	11 (4.6)	6 (2.5)	10 (4.2)
Extremely severe	5 (2)	10 (4.2)	12 (5)	11 (4.6)	2 (0.8)	5 (2.1)

## Discussion

To the best of our knowledge, this is the first study that has evaluated the measurement invariance of the DASS-21 across male and female medical students. Since clinical decisions about psychological intervention are frequently made on the basis of the results of psychological assessment tools, it is necessary to know whether these instruments function similarly across people with different backgrounds. This study represents the DASS-21 as a screening instrument to consider that depression, anxiety, and stress have an acceptable internal consistency as well as excellent convergent and construct validity in Iranian medical students. The CCFA results provide support in this regard to conclude that the three subscales of the DASS-21 predominantly capture their intended psychological constructs as a whole and in both male and female medical students. Moreover, mean item-correlation for each subscale of the DASS-21 were between 0.20 and 0.41, showing that while the items in each subscale are rationally homogenous, they are not isomorphic (i.e., not exactly identical or similar in form and content). 

The results of DIF analysis also showed that DASS-21 is an invariant measure across genders in medical students and it can be used for meaningful comparison of depression, anxiety and stress scores between medical student genders. Our findings revealed that, except just one item in the stress subscale, male and female medical students respond consistently to the items in the DASS-21 instrument. In order to know to what extent this item can distort group differences on the target subscale, we removed Item 6: “I was intolerant of anything that kept me from getting on with what I was doing” with non-uniform DIF from the stress domain. Although removing it from the stress subscale specifically affected the mean scores of the male and female groups given in Table 4, the findings did not change principally. This means that with or without inclusion of Item 6, the stress mean score was not statistically significant across male and female medical students.

Any comparison of means between male and female medical students could be problematic if we do not assess measurement invariance. Hence, in case of the present study, findings of no difference in subscale scores across genders ensure the absence of real differences and it is not a result of systematic bias in response patterns or different interpretations of the questions by male and female medical students. Moreover, our sample size is relatively large and hence the lack of significant differences in terms of gender in the mean scores of the three subscales cannot be attributed to the sample size.

The findings of the present study provide a new insight into the role of gender and distress measures in shaping medical education. Having the same perception of the concept of stress, anxiety, and depression at the item and scale levels of the DASS-21 instrument indicates that the academic performance of male and female Iranian medical students can be equally influenced by distress measures. However, gender distress similarities across male and female medical students may be attributed to the highly selective nature of the homogeneous sample of students from one medical school in Southern Iran.   

As this is the first study organized to evaluate the measurement invariance of the DASS-21 across male and female medical students, there was no comparable research in the literature. However, despite the use of different statistical methods, our findings were in line with three previous studies, demonstrating that the BDI, GHQ-12, and CSSS instruments were invariance across male and female non-medical students.^39^^-^^41^ In general, if we intend to draw one general conclusion by linking the findings of our current research with the three previous studies, it would be that male and female students perceive the meaning of items in the DASS-21, BDI, GHQ-12, and CSSS in a consistent manner. Moreover, differential item functioning analysis in a previous study revealed that the items in the DASS-21 function similarly across male and female with chronic low back pain.^38^ However, our findings were different from those of the previous research, which provided evidence for the lack of measurement invariance of the DASS-21 across racial groups in the United States.^29^ The possible explanations for such differences may be due to the different statistical methods and samples employed for invariance testing.

Our findings were consistent with those of previous studies in Iran,^2^ India^17^ and Saudi Arabia,^11^ which showed no differences in the mean stress scores between male and female preclinical medical students. Although a previous study reported a high level of stress (60%) among Iranian medical students,^2^our findings revealed that the rate of stress (mild to extremely severe) is approximately 35% in each gender group. These differences in findings can be attributed to different questionnaires used in these studies. While we used the DASS-21 to assess stress, the two aforementioned studies in Iran applied the Kessler 10-item.

Our study also has a number of limitations that need to be mentioned. Depression, anxiety, and stress were determined by the DASS-21 as a self-assessment measure, and no objective clinical assessment was conducted to confirm whether students were actually suffering from distress. Another limitation is that the present research is a cross-sectional survey, and a longitudinal study is needed to explore how distress in medical students changes through the course of schooling. A previous longitudinal study has shown that anxiety scores change during medical training; however, it reported no difference in depression scores by gender.^49^

## Conclusions

This is the first study that has evaluated measurement invariance of DASS-21 across medical student genders. The present research revealed that male and female Iranian medical students perceived and interpreted the meaning of almost all the DASS-21 items in a similar manner. Accordingly, DASS-21 can be used as an invariant measure for meaningful comparison of depression, anxiety and stress scores across medical student genders. In the present study, no differences in the subscale scores across genders ensure the absence of real differences and do not reflect an artificial effect relating to different interpretations of items by genders in medical students. Future research should attempt to move on from the cross-sectional study to longitudinal work to test the hypothesis, which cannot be explored with simple cross-sectional data. As detecting DIF may vary substantially from one measure to another,^50^^-^^53^ future studies should focus on assessing DIF across male and female medical students by other psychological instruments. Moreover, future DIF studies should include additional populations that vary in culture, race, and ethnicity, in addition to years in college and college major. Finally, the performance of the DASS-21 should be examined for agreement with clinician judgement on the basis of a structured diagnostic interview such as the Mini International Neuropsychiatric Interview.

### Acknowledgments

This work was supported by the grant number 93-01-21-8075 from Shiraz University of Medical Sciences Research Council, Shiraz, Iran. This article was extracted from Farnoosh Nozari’s MD thesis.

### Conflicts of Interest

The authors declare that they have no conflict of interest.
